# Large number of phosphotransferase genes in the *Clostridium beijerinckii* NCIMB 8052 genome and the study on their evolution

**DOI:** 10.1186/1471-2105-11-S11-S9

**Published:** 2010-12-14

**Authors:** Yixiang Shi, Yi-Xue Li, Yuan-Yuan Li

**Affiliations:** 1 Bioinformatics Center, Key Laboratory of Systems Biology, Shanghai Institutes for Biological Sciences, Chinese Academy of Sciences, Shanghai 200031, P.R. China; 2 Shanghai Center for Bioinformation Technology, Shanghai 200235, P.R. China

## Abstract

**Background:**

*Clostridium beijerinckii* is a valuable bacteria species which has the ability of ABE (acetone, butanol and ethanol) production. It has been shown that Phosphotransferase (PTS) is an important and common system for both carbohydrate uptake and phosphorylation in bacteria, but detailed study of the system, especially its fructose/mannose/sorbose family is scant.

**Results:**

In the genome of *Clostridium beijerinckii* NCIMB 8052, a model strain recently sequenced, there are large number of PTS genes, among them 9 complete sets belong to the fructose/mannose/sorbose family of its enzyme II complex. Our study, based on evidences provided by phylogenetic relationship, analyses of gene contents and clusters, as well as synteny examination, indicates that it is possible to further classify this PTS family into three sub-groups, which are corresponding to the three sugar substrates. Furthermore, we proposed a model how these PTS systems are evolved in bacteria.

**Conclusion:**

This work may explain the experimental result that *Clostridium beijerinckii* NCIMB 8052 can better utilize fructose as substrate, thus could lead to a better understanding of the ABE-producing mechanism in *Clostridium beijerinckii* and other microbial species. It may help to illustrate a higher butanol-productivity future.

## Background

**Figure 1:**
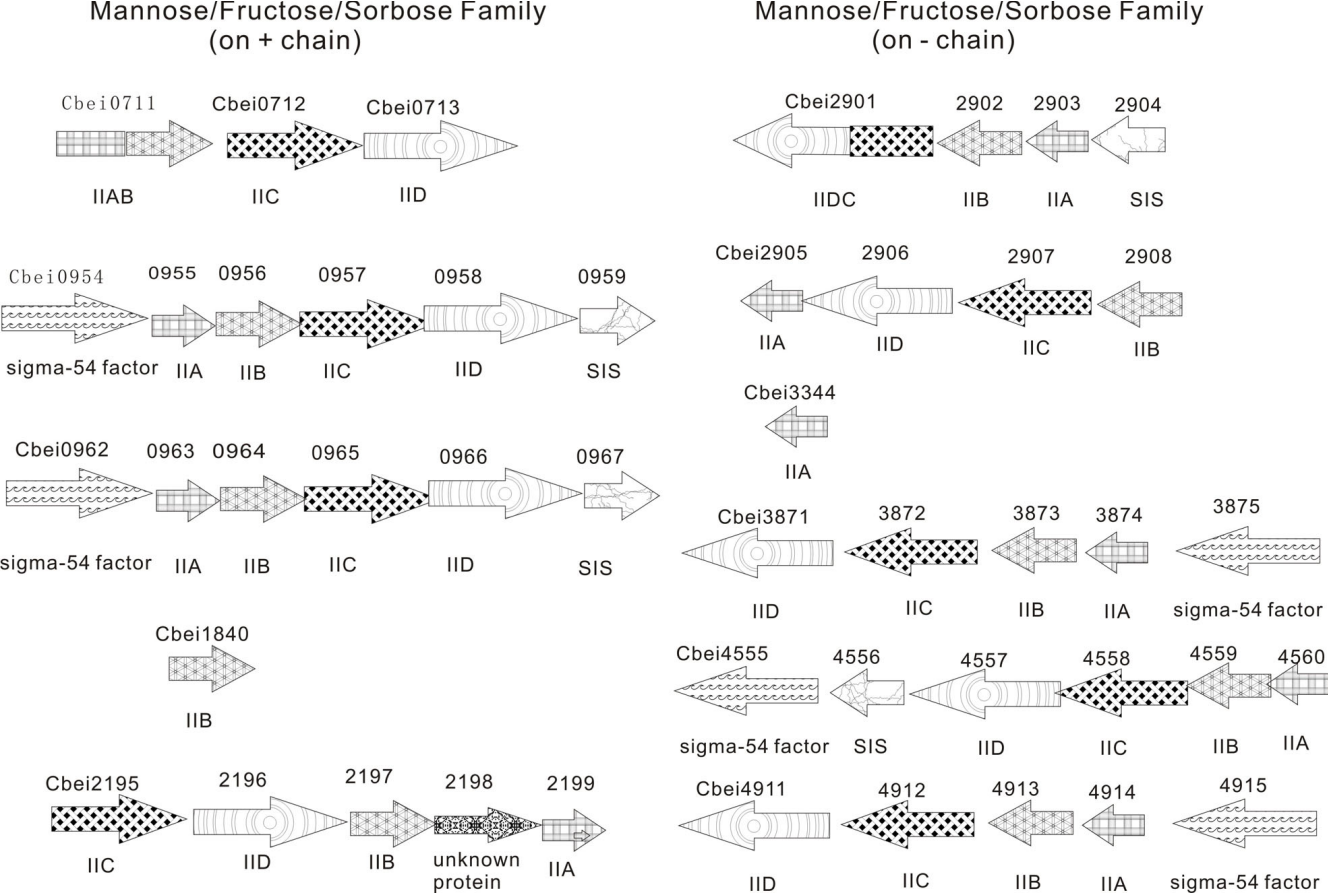
The genomic organization of *Clostridium beijerinckii* NCIMB 8052 genes encoding phosphotransferases of fructose/mannose/sorbose family. Left panel: the fructose/mannose/sorbose family of the PTS II genes on the positive chain; Right panel: the fructose/mannose/sorbose family of the PTS II genes on the negative chain.

The anaerobic Gram-positive bacterium *Clostridium beijerinckii* is capable of producing acetone, butanol and ethanol (which are together called ABE)[1]. These three are important chemical products, especially butanol, since it is not only a food-grade solvent, but also an excellent fuel potentially could be used as a replacement for oil. Its features such as high boiling point and low emission are quite desirable [1]. Similar to another ABE-producing model strain *Clostridium acetobutylicum* ATCC 824, the ratio of the solvent produced by *Clostridium beijerinckii* is close to 3:6:1 (A:B:E, v/v/v) [1,2]. But *Clostridium beijerinckii* has the advantage that its fermentation does not require the using of starch, which is a food source. It can utilize various kinds of biomass, including ‘waste’ material, therefore can lower the producing cost and enhance its practical competitiveness [1,3]. Because of its great industrial potential, its model strain *Clostridium beijerinckii* NCIMB 8052 has been sequenced by JGI (Joint Genome Institute, DOE, USA) and the genomic data were made public in 2007 (http://genome.jgi-psf.org/clobe/clobe.home.html). The ABE-producing process starts from pyruvate, the end product of glycolysis. Pyruvate can be further degraded into acetyl-CoA. Acetyl-CoA could be transformed into ethanol, or through several steps, into butanol and acetone (see Figure 1 of the reference [4]). So, the more monosaccharide molecules which are transferred into cells, and the higher sugar utilization efficiency, the better for the ABE-production. Phosphotransferase system (PTS) is an important mechanism for sugar transmembrane transportation and phosphorylation in bacteria [5-7]. This system is consisted of enzyme I, phosphocarrier protein (Hpr) and enzyme II complex. Enzyme I and Hpr will phosphorylate enzyme II, which is also called permease since it is the unit which carries out the transmembrane transportation. Enzyme II has A, B, C, D subunits. In some microbial species these subunits each exist as a separate peptide, but in some other cases they may fuse together in various combinations. The IIB subunit is the one which decides the substrate specificity. Enzyme II can be classified into at least 7 families [8], according to their specific sugar substrates, and in different families the complex may be organized in different ways [5]. Among them, the fructose/mannose/sorbose family is the only one which has IID subunit, and all the members in this family are capable of transport fructose [6]. The most well known genes in this family, include fructose-specific ones in *Bacillus subtilis*[9], mannose-specific genes in *Escherichia coli*[10] and sorbose-specific genes in *Klebsiella pneumoniae*[11].

Previous studies have revealed that enzyme I and the phosphocarrier protein (Hpr) of PTS generally has only one or two copies in clostridium strains, and they exist in the genomes monocistronically [12]. However, the genes consisted of the enzyme II complex may have multiple copies, and often form operons together with their transcription regulatory units and the genes with related catalytical functions [13]. Furthermore, the phosphotransferase systems in different types of microbial species have different mechanisms for regulation. For low GC Gram-positive bacteria like clostridium, Hpr is acting as an important switch. When carbon source is in short supply, Hpr will be phosphorylated on the serine-46 residue by certain unique Hpr kinase/phosphatase. In contrast, if carbon source is sufficient, the His-15 site of Hpr is phosphorylated, and the PTS-dependent sugar transportation and substrate-specific regulation are activated [14].

Although the study on phosphotransferase system has been going on for several decades, most of the previous reports are concentrated on limited number of model strains such as *Escherichia coli* and *Bacillus subtilis*. Since the beginning of this century, there are some researches on PTS in clostridium, mostly on *Clostridium acetobutylicum* ATCC 824, which is the first clostridium sequenced. The importance of PTS to the life process of various clostridium strains, and how the system is evolved, remain largely unknown. This work starts from the genomic data of *Clostridium beijerinckii* NCIMB 8052, and other sequenced clostridium strains as well as some important related model species, and analyzes the complexity of the phosphotransferase system, especially focusing on the fructose/mannose/sorbose family. We hope to explain why and how *Clostridium beijerinckii* NCIMB 8052 can utilize more types of sugar substrate, and propose an evolutionary model for the phosphotransferase system. It may help to illustrate the solvent-producing mechanism in *Clostridium beijerinckii* and other related industrial bacteria, which could have theoretical and practical importance for guiding to higher butanol production through certain optimizations.

## Results and discussion

### Large number of PTS II genes are identified in the *Clostridium beijerinckii* NCIMB 8052 genome

Our study finds that there are 47 sets of PTS II genes in the *Clostridium beijerinckii* NCIMB 8052 genome (See Additional File 1, Table S1). That is far more than the majority of the other sequenced clostridium strains, including *Clostridium botulinum* A str. ATCC 3502 [12], *Clostridium acetobutylicum* ATCC 824 (Table S2), *Clostridium perfringens* strain 13 [15] (Table S3), *Clostridium tetani* E88 [16] (Table S4). Only *Clostridium difficile* 630 [17] (Table S5) has a similar number. The PTS II gene copies in other species which are evolutionarily closer to clostridium are also significantly lower than that of *Clostridium beijerinckii* NCIMB 8052 genome. For example, *Bacillus subtilis* (Table S6) has 12 sets. These findings are summarized in Table 1. There are 9 complete sets of PTS II genes which are belonging to the fructose/mannose/sorbose family among the 47 ones found in the *Clostridium beijerinckii* NCIMB 8052 genome, plus one additional IIA and IIB genes each (Table 2). Such a large number is never reported in any other known microbial species, including *Clostridium difficile* 630 (additional file 1). Their genomic organizations are illustrated in Figure 1. Interestingly, all the fructose/mannose/sorbose family PTS II genes on the positive chain are located on the first half of the genome, while the ones on the negative chain are on the second half.

**Table 1:** The bacterial strains studied and the sets of the PTS II genes in their genomes

Bacteria/strain	Sets of PTS II genes
*Clostridium beijerinckii* NCIMB 8052	47
*Clostridium botulinum* A str. ATCC 3502	15
*Clostridium acetobutylicum* ATCC 824	14
*Clostridium perfringens* strain 13	14
*Clostridium tetani* E88	2
*Clostridium difficile* 630	49
*Bacillus subtilis* subsp. *subtilis* str. 168	12
*Escherichia coli* str. *K-12* substr. DH10B	25

In contrast, the PTS enzyme I and the phosphocarrier protein Hpr, each have only one copy in the *Clostridium beijerinckii* NCIMB 8052 genome (Cbei0196 and Cbei1219, respectively). This is consistent with other clostridium species.

### Analysis of GC content

To study these sets of fructose/mannose/sorbose family PTS II genes in *Clostridium beijerinckii* NCIMB 8052 in detail, we first calculated their GC percentages (Table 2).

**Table 2:** The GC distribution of the fructose/mannose/sorbose PTS II gene sets in *Clostridium beijerinckii* NCIMB 8052. Three groups are indicated, with *, #, or nothing at all.

Cbei Number	Domain Structure	GC%
0711/0712/0713	IIAB/IIC/IID	37.5
0955/0956/0957/0958*	IIA/IIB/IIC/IID	31.8
0963/0964/0965/0966*	IIA/IIB/IIC/IID	31.9
1840#	IIB	30.8
2195/2196/2197/2199*	IIC/IID/IIB/IIA	33.9
2901/2902/2903*	IICD/IIB/IIA	32.4
2905/2906/2907/2908*	IIA/IID/IIC/IIB	32.0
3344*	IIA	31.7
3871/3872/3873/3874#	IID/IIC/IIB/IIA	35.1
4557/4558/4559/4560*	IID/IIC/IIB/IIA	30.3
4911/4912/4913/4914#	IID/IIC/IIB/IIA	35.1

As we can see from the result, the GC contents of these sets of fructose/mannose/sorbose family PTS II genes can be divided into three groups, which are indicated in the Table 2 with three different denotations, with *, # or nothing at all.

For the two sets of Cbei3871/3872/3873/3874 and Cbei4911/4912/4913/4914, both their GC percentages are 35.1%. The GC% of Cbei0711/0712/0713 is over 37%. For the remaining sets, their GC percentages are ranging from 30.3% to 33.1%. Although that of Cbei2195/2196/2197/2199 is 33.9%, but if the inserted hypothetical protein Cbei2198 is considered altogether, it drops to 33.1%. The two orphan genes of this family, Cbei1840 and Cbei3344, are not necessarily conforming to this rule. It is noteworthy that the GC percentage of the *Clostridium beijerinckii* NCIMB 8052 whole genome is only 29.9%, lower than that of any PTS II genes sets. We will discuss this later.

### Synteny analysis

According to Figure 1, the fructose/mannose/sorbose family PTS II genes in *Clostridium beijerinckii* NCIMB 8052 can also be classified into three groups based on their synteny features. One group has both sigma-54 factor and sugar isomerase (SIS) in the neighborhood, such as Cbei0955/0956/0957/0958, Cbei0963/0964/0965/0966 and Cbei4557/4558/4559/4560. Another group has sigma-54 factor only, including Cbei3871/3872/3873/3874 and Cbei4911/4912/4913/4914. The last group has neither sigma-54 factor nor sugar isomerase, like Cbei0711/0712/0713. We found these groupings are quite consistent with the ones discussed in the last section which are based on their GC percentages.

Sigma-54 is a kind of transcriptional factors, commonly existed in bacteria. It can interact with promoters to activate the transcription process. The first sigma-54 factor, rpoN, was found in 1985 through the sequencing of *Klebsiella pneumoniae*[18]. According to previous report [19] , in *Listeria monocytogenes*, sigma-54 factor-dependent fructose/mannose/sorbose PTS II genes are found to be associated with the bacteria’s sensitivity of cytotoxin. Therefore, it is reasonable to suggest that their presence around the fructose/mannose/sorbose PTS II genes in *Clostridium beijerinckii* NCIMB 8052, could also have certain functional importance.

### Construction of phylogenetic tree

Since IIB is the subunit which decides the PTS substrate specificity, in this part of study we use Cbei3873 as the query for blastp, and analyze the evolutionary relationship among the PTS fructose/mannose/sorbose family IIB genes in *Clostridium beijerinckii* NCIMB 8052 and their homologous genes identified from other representative bacteria species (selecting according to the e-value so that every e-value intervals have representations).

**Figure 2:**
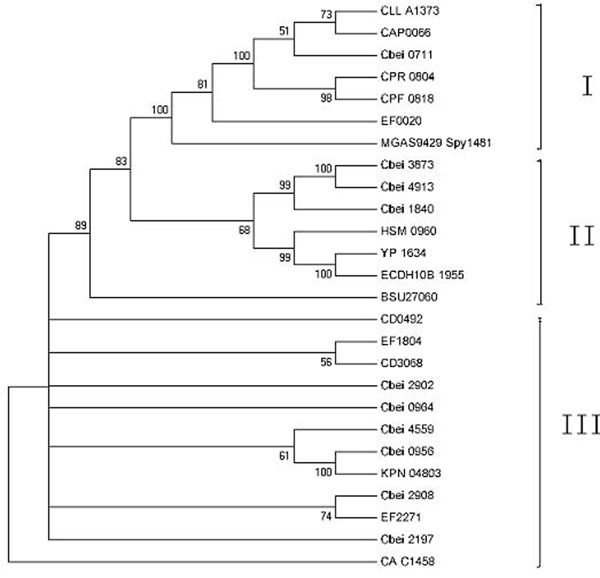
The phylogenetic tree built using the PTS IIB genes of fructose/mannose/sorbose family in *Clostridium beijerinckii* NCIMB 8052 and some representative homologs found in other bacteria.

As we can see from the phylogenetic tree in Figure 2, the PTS fructose/mannose/sorbose family IIB genes in *Clostridium beijerinckii* NCIMB 8052 can be grouped into three clusters too. One cluster contains Cbei0711, another includes Cbei1840, Cbei3873 and Cbei4913, while Cbei0956, Cbei0964, Cbei2902 and Cbei4559 are together in the other. Especially noteworthy is that the third cluster includes the known sorbose-specific IIB gene KPN_04803 from *Klebsiella pneumoniae*.

Among the group which contains Cbei1840, Cbei3873 and Cbei4913, Cbei3873 is 95% identical to Cbei4913, 75% identical to Cbei1840, and 39% identical to the fructose-specific IIB gene Bsu27060 in *Bacillus subtilis* over 160 aa (68% positive) (Figure 2). However, Cbei3873 has also been found to be 59% identical to the mannose-specific IIAB gene Ecoli_UTI89_C2014. On the other hand, although Cbei0711 is the closest to Ecoli_UTI89_C2014 in terms of gene organization since in both cases the IIA and IIB subunits are fused together, they are not grouped in the same cluster.

This result reveals the complexity of the PTS fructose/mannose/sorbose family. The reason that the transport systems for these three sugars can be classified into one family is because they are close to each other, highly homologous to each other, with few homologies to the genes in other families. But looking into it in more detail, we can find that PTS II genes for fructose and mannose transportation are closer than their relation with that of sorbose. As the result, both fructose and mannose PTS II genes phosphorylate fructose into phospho-6-fructose (F-6-P), while sorbose PTS II genes turns it into F-1-P. Furthermore, according to reference [5], the phosphorylation site on the fructose by the fructose-specific PTS genes is O-6, instead of the more common O-1 site. This is another proof that fructose-specific PTS genes are closer to the mannose-specific ones.

### An evolutionary model is proposed

To get a clearer picture of why there are so many copies of fructose/mannose/sorbose family PTS II genes in the *Clostridium beijerinckii* NCIMB 8052 genome, we applied the same analytical approaches to the model strain of *Bacillus subtilis*, *B. subtilis* subsp. subtilis str. 168, by identifying all the PTS II genes in its genome, annotating and classifying each sets as well as calculating their GC percentages. The results are in Table S6 and showed that there are 12 sets of PTS II genes in *B. subtilis* subsp. subtilis str. 168. One of them belongs to the fructose/mannose/sorbose family, namely Bsu27040/27050/27050/27070, which phosphorylates fructose into F-6-P. The GC percentages of these PTS II gene sets in *B. subtilis* subsp. subtilis str. 168 are ranging from 44.4% to 50.8%, with that of Bsu27040/27050/27050/27070 is 44.4%, slightly higher than the GC percentage of the whole genome, which is 43.5%.

We did the same analysis to the model strain of *E. coli*, *Escherichia coli* str. K-12 substr. DH10B (Table S7). 25 sets of PTS II genes are identified, including one complete set of the fructose/mannose/sorbose family, i.e., ECDH10B 1955/1956/1957. Their GC contents are as high as 56.1%, and as low as 47.3%, while that of ECDH10B 1955/1956/1957 is 51.6%, close to the average GC percentage of the *Escherichia coli* str. K-12 substr. DH10B genome, 50.8%.

As the results indicate, in all these bacterial species we studied, the GC percentages of PTS II gene sets are generally higher than the overall GC values of the host genomes. This leads us to speculate that the phosphotransferase system may have originated from certain high GC percentage microbial species, and during the evolutionary process lower GC percentage species acquired the components of this system from higher GC percentage species through horizontal gene transfer (HGT). The transfer may occur at an earlier stage of the evolution, since it becomes clear that the GC percentages of PTS II gene sets are being drawing closer to the average values of the host species after the transfer. For example, in *Escherichia coli* str. K-12 substr. DH10B, they are generally between 50-60%. The values drop to the interval of 45-50% in *B. subtilis* subsp. subtilis str. 168. In the clostridium strains in which GC contents are even lower, the values further decrease. In the case of *Clostridium beijerinckii* NCIMB 8052, they are 30 to 38%. Of course, this makes sense. Understandably, the sugar transport system is of the utmost importance to bacteria, concerning the life or death of each organism. So this system must have existed in microbial species for quite a long time. However, as two strains highly representative of the fructose/mannose/sorbose family, both the fructose-specific PTS II genes in *Bacillus subtilis*, and the mannose-specific ones in *E. coli*, their GC percentages are closer to the overall GC contents of their genomes. Therefore, they may be ‘native’ to these species and later on transferred into *Clostridium beijerinckii* NCIMB 8052.

**Figure 3:**
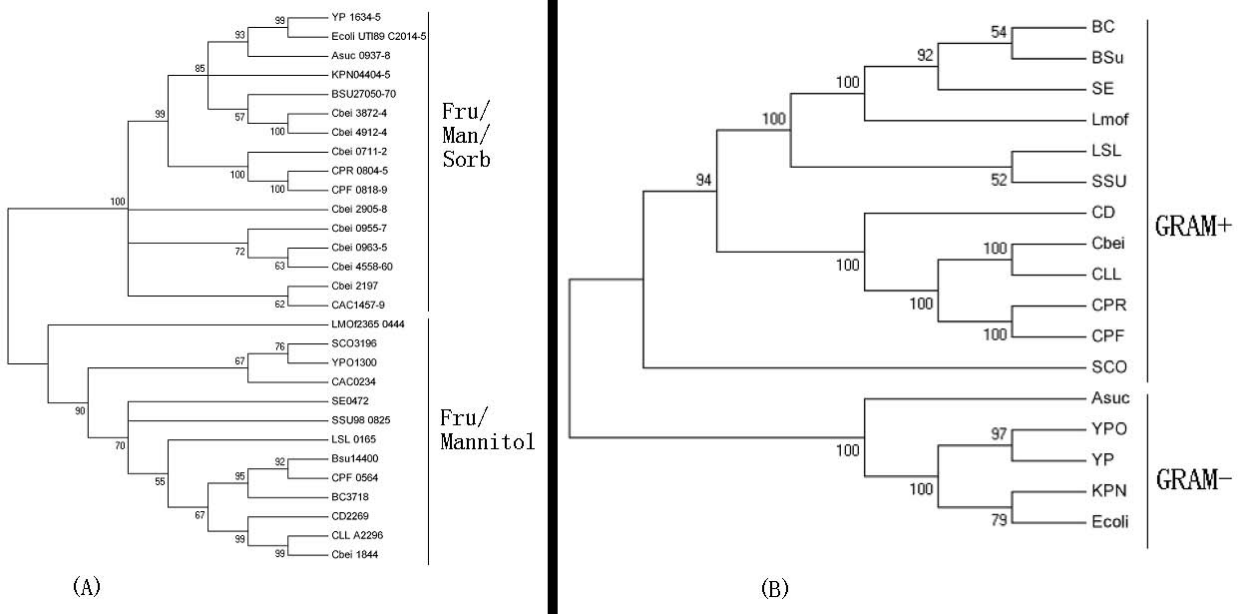
The Evolutionary model of the microbial PTS II system is proposed. (A) the phylogenetic tree built by using fructose/mannose/sorbose and fructose/mannitol families of the PTS II ABC genes; (B) the 16sRNA tree of the studied microbial species.

To test our hypothesis that lower GC content microbial species obtained PTS II genes from higher GC ones during the evolutionary process through horizontal gene transfer, we introduce PTS II genes from the fructose/Mannitol [20] family as references (use Cbei1844 as the query for blastp, since it has been confirmed as a member). This family, which contains no IID subunit, can phosphorylate fructose into F-1-P. Like the case of Cbei1844, in a lot of species, the IIABC subunits are fused together as one gene. Since there is no IID subunit for this fructose/mannitol family, we use representative IIABC genes from the fructose/mannose/sorbose and fructose/mannitol families, run blastp separately, and use selected (selecting according to the e-value so that every e-value intervals have representations) resulting genes from both to construct another phylogenetic tree (Figure 3A). We also construct the 16sRNA tree of the species involved (Figure 3B). As shown in the 16sRNA tree, these studied species fall into two groups. *Escherichia coli* UTI89, *Klebsiella* pneumoniae subsp. *pneumoniae* MGH 78578 (KPN), *Yersinia pestis* CO92 (YPO), *Yersinia pestis* biovar Microtus str. 91001 (YP) and *Actinobacillus succinogenes* 130Z (ASuc) are grouped together (lower half of Fig. 3B). They are all Gram-negative strains with relatively higher GC contents. Another group contains all kinds of clostridium, *Bacillus subtilis* subsp. subtilis str. 168, *Staphylococcus epidermidis* ATCC 12228 (SE), *Streptococcus suis* 98HAH33 (SSU), *Streptomyces coelicolor* A3(2) (SCO), *Listeria monocytogenes* str. 4b F2365 (LMof) and *Lactobacillus salivarius* (LSL), which are all Gram-positive strains. The phylogenetic tree in Fig. 3A also includes two main branches. The upper one is the PTS II genes from the fructose/mannose/sorbose family, while genes from the fructose/mannitol family are in the lower half. Comparing the trees in Figs. 3A and 3B, we can find that the PTS II genes from *Bacillus subtilis* and clostridium strains are grouped together with those from the Gram-negative strains which we identified as the lower half in Fig. 3B. This supports our hypothesis.

### How the PTS II systems are evolved in *Clostridium beijerinckii* NCIMB 8052?

The genome size of *Clostridium beijerinckii* NCIMB 8052 is about 5.9MB, including 5104 ORFs. That is significantly larger than that of *Clostridium acetobutylicum* ATCC 824, which has a chromosome of 3.94MB plus a plasmid of 192KB [2], totaling 3917 ORFs (3739 on the chromosome and 178 on the plasmid pSol1). This is likely due to more diverse living environments enjoyed by *Clostridium beijerinckii* NCIMB 8052, which means more types of carbon sources it can utilize [1,3]. Although they are in the same genus of clostridium, they separated relatively early in the evolutionary process [21]. *Clostridium acetobutylicum* ATCC 824 has been shown acquiring large quantities of outside genes through horizontal gene transfer [2]; *Clostridium difficile* 630 also has been found with as high as 11% of mobile gene contents in its genome [17], which strongly indicates that it gained genes from other species. So, it is reasonable to expect similar events happened in *C. beijerinckii* NCIMB 8052 too. But in its distintive evolutionary process, *C. beijerinckii* NCIMB 8052 is able to keep large number of genes transferred from other species because it needs these genes to adapt to various soil environments. On the other hand, the living condition of *C. acetobutylicum* ATCC 824 is relatively simpler, so only fewer genes which are essential to its life activity are kept. More specifically, *C. beijerinckii* NCIMB 8052 is quite different from *C. acetobutylicum* ATCC 824 in terms of sugar needed. While *C. acetobutylicum* ATCC 824 is starch-based, *C. beijerinckii* NCIMB 8052 is molasses-based. Molasses contains 35% of sucrose, 7% of fructose and 5% of glucose (W. Jiang, personal communication). Also, *C. beijerinckii* NCIMB 8052 can ferment xylose degraded from agricultural wastes [22]. Since *C. beijerinckii* NCIMB 8052 can utilize more sugar substrates than *C. acetobutylicum* ATCC 824, it makes senses that it needs more genes to break down these substrates. After querying the ISFinder database (http://www-is.biotoul.fr/), we identified 92 different types of insertion sequences in the *C. beijerinckii* NCIMB 8052 genome, among them 19 with e-score lower than 1e-5 (data not shown). A lot of these insertion sequences have multiple copies. For example, ISCb1[23] has 9 copies. In *Clostridium perfringens*, some PTS genes are found to be flanked by insertion sequences [24]. In *C. beijerinckii* NCIMB 8052, we have similar findings too. This is in agreement with our hypothesis that *C. beijerinckii* NCIMB 8052 genome gained and kept large quantities of outside genes through HGT, including some PTS genes.

Another reason which leads to the fact that there are so many sets of the PTS II genes in the *C*. *beijerinckii* NCIMB 8052 genome is gene duplication. As shown in Figure 2, many PTS IIB genes have several close-related copies. For example, Cbei3873 and Cbei4913, they are 95% identical. Figure 1 shows that they share the same genetic organization and gene order. This strongly indicates that these genes are of great importance to the host bacteria, so they are duplicated in group, as functional backups for the original ones.

### The correlation between sugar utilization and PTS II gene numbers

*Clostridium beijerinckii* NCIMB 8052 can better utilize fructose in the fermentation media than *Clostridium acetobutylicum* ATCC 824 (W. Jiang, personal communication). Our findings have shown that in the *Clostridium beijerinckii* NCIMB 8052 genome there are so many more gene sets of PTS II fructose/mannose/sorbose family than C. *acetobutylicum* ATCC 824. And with the consensus from previous reports [6] that all gene sets of this family can transport fructose, we established a likely correlation between these facts.

In addition to the PTS system, fructokinase [25] can also phosphorylate fructose into phospho-6-fructose. However, fructokinase may not have played a major role in determining the difference between the fructose utilization efficiencies of these two clostridium model strains. In both genomes, there are two copies of fructokinase genes, Cbei3497 and Cbei5009 ;CAC0424 and CAC1523.

It remains an unsolved question why these three sugars, fructose, mannose and sorbose, can form such a distinctive phosphotransferase family. In terms of their chemical structures, all three are hexose, but fructose and sorbose are ketoses while mannose is an aldose. Probably they have very similar 3D structures. The presence of sugar isomerases around certain types of the fructose/mannose/sorbose family PTS II genes in the *C. beijerinckii* NCIMB 8052 genome seems to suggest this too.

## Conclusions

In summation of the analysis performed in this research , we found that the fructose/mannose/sorbose family of PTS II genes in *Clostridium beijerinckii* NCIMB 8052 can be further classified into three groups , possibly corresponding to the three different sugar substrates of this family. In addition to their respective specific sugars, all of them are capable of transport fructose. It is highly likely that this finding offers a valid explanation that *Clostridium beijerinckii* NCIMB 8052 can better utilize fructose as the sole carbon source than *Clostridium acetobutylicum* ATCC 824.

We also proposed an evolutionary model which suggests the horizontal gene transfer of the phosphotransferase system from higher GC microbial to lower GC ones. This may help to illustrate the mechanism why bacteria can survive in different environments through their variable carbon sources.

## Methods

### Homology search

The amino acid sequences of the studied genes are used as query, run blastp search [26] against the non-redundant (nr) database ,keep all the default parameters values, and find their homologous genes in other sequenced microbial species. E-value should be less than 1e-5.

### Annotation of the PTS II genes

All three methods listed below are used for the accurate annotation of the genes in the *Clostridium beijerinckii* NCIMB 8052 genome and other studied genomes, which are downloaded from the NCBI (http://www.ncbi.nlm.nih.gov/) RefSeq database.

(1) Blastp: described above.

(2) InterproScan [27] domain analysis: the amino acid sequences are submitted to the EBI website of InterProScan (http://www.ebi.ac.uk/Tools/InterProScan/), using all the default parameter values.

(3) The domain structures of the PTS II genes are compared with those known variations of all the families from the literatures.

### Synteny analysis

For each sets of the PTS II genes, we first visually inspect their downstream and upstream regions for possible existence of synteny. The amino acid sequences of the 4 surrounding genes in both directions are picked out to identify their domains through InterproScan. They are used as the blastp queries to identify their homologous genes in other microbial species (e-value less than 1e-5). We then check the locations of these homologous genes in their respective genomes to see whether they also appear in the surrounding areas of PTS II genes.

### Multi-sequence alignment and construction of phylogenetic trees

We use the embedded ClustalW [28] function of software package MEGA4 [29] for multisequence alignment, using all default parameter values. NJ (Neighbor-Joining) method [30] is applied to construct phylogenetic trees, with bootstrap values [31] calculated by repeating 500 times to evaluate the robustness of the nodes on the trees.

## Competing interests

The authors declare that they have no competing interests.

## Authors' contributions

YS designed the study and performed the analyses and interpret the results. YS and YYL drafted the manuscript. YXL supervised the study.

## Supplementary Material

Additional file 1 We surveyed all the PTS genes in 7 species and listed their domain structure, predicted families as well as the GC percentage for each sets. Click here for file
