# Supplementary material for: Large number of phosphotransferase genes in the Clostridium beijerinckii NCIMB 8052 genome and the study on their evolution
Source: BMC Bioinformatics. 2010 Dec 14;11(Suppl 11):S9. doi: 10.1186/1471-2105-11-S11-S9 (PMC3024867; doi:10.1186/1471-2105-11-S11-S9)
Supplement: Additional file 1 — We surveyed all the PTS genes in 7 species and listed their domain structure, predicted families as well as the GC percentage for each sets. [file 1471-2105-11-S11-S9-S1.doc]

**Supplementary Material**

Table S1 the PTS II gene sets in *Clostridium beijerinckii* NCIMB 8052

| Cbei Number | Domain Structure | Subfamily | GC% |
| --- | --- | --- | --- |
| 0220/0221/0222 | IIA/IIB/IIC | lactose/cellobiose | 32.9 |
| 0242/0244 | IIBC/IIA | Mannitol/fructose | 33.9 |
| 0336/0337/0339 | IIC/IIBC/IIA | glucitol/sorbitol | 33.7 |
| 0380 | IIA | lactose/cellobiose | 30.1 |
| 0525/0527 | IIA/IIBC | Mannitol/fructose | 34.2 |
| 0541/0542/0543 | IIA/IIB/IIC | lactose/cellobiose | 34.3 |
| 0699 | IIBC | lactose/cellobiose | 34.4 |
| 0711/0712/0713 | IIAB/IIC/IID | mannose/fructose/sorbose | 37.5 |
| 0751 | IIABC | N-acetylglucosamine/Glucose | 35.3 |
| 0758 | IIB | lactose/cellobiose | 33.5 |
| 0950/0951/0952 | IIC/IIB/IIA | lactose/cellobiose | 32.3 |
| 0955/0956/0957/0958 | IIA/IIB/IIC/IID | mannose/fructose/sorbose | 31.8 |
| 0963/0964/0965/0966 | IIA/IIB/IIC/IID | mannose/fructose/sorbose | 31.9 |
| 1475/1476/1478 | IIA/IIB/IIC | lactose/cellobiose | 34.4 |
| 1840 | IIB | mannose/fructose/sorbose | 30.8 |
| 1844 | IIABC | Fructose/mannitol | 35.7 |
| 1918 | IIBC | Unknown* | 34.1 |
| 2195/2196/2197/2199 | IIC/IID/IIB/IIA | mannose/fructose/sorbose | 33.9 |
| 2320 | IIBC | N-acetylglucosamine/Glucose | 35.2 |
| 2498/2499/2500 | IIA/IIB/IIC | lactose/cellobiose | 33.6 |
| 2663/2665/2666 | IIA/IIC/IIB | lactose/cellobiose | 34.3 |
| 2707/2708/2709 | IIC/IIB/IIA | lactose/cellobiose | 33.3 |
| 2739/2740/2741 | IIC/IIB/IIA | lactose/cellobiose | 32.8 |
| 2833 | IIABC | beta-glucoside/sucrose | 32.4 |
| 2901/2902/2903 | IICD/IIB/IIA | mannose/fructose/sorbose | 32.4 |
| 2905/2906/2907/2908 | IIA/IID/IIC/IIB | mannose/fructose/sorbose | 32.0 |
| 3273 | IIABC | beta-glucoside/sucrose | 33.4 |
| 3344 | IIA | mannose/fructose/sorbose | 31.7 |
| 3768/3772 | IIA/IIBC | N-acetylglucosamine/Glucose | 33.6 |
| 3811/3812/3813 | IIA/IIB/IIC | lactose/cellobiose | 32.1 |
| 3871/3872/3873/3874 | IID/IIC/IIB/IIA | mannose/fructose/sorbose | 35.1 |
| 4158 | IIB | Unknown | 28.4 |
| 4532/4533 | IIBC/IIA | N-acetylglucosamine/Glucose | 33.8 |
| 4535/4537/4538 | IIC/IIA/IIB | lactose/cellobiose | 31.8 |
| 4557/4558/4559/4560 | IID/IIC/IIB/IIA | mannose/fructose/sorbose | 30.3 |
| 4634/4635/4636 | IIC/IIB/IIA | lactose/cellobiose | 34.3 |
| 4638/4639/4640 | IIC/IIB/IIA | lactose/cellobiose | 33.6 |
| 4683/4684/4685 | IIC/IIB/IIA | lactose/cellobiose | 32.2 |
| 4705/4706 | IIBC/IIA | N-acetylglucosamine/Glucose | 33.6 |
| 4804/4806 | IIA/IIBC | N-acetylglucosamine/Glucose | 32.4 |
| 4838 | IIABC | N-acetylglucosamine/Glucose | 33.1 |
| 4846 | IIABC | beta-glucoside/sucrose | 33.6 |
| 4911/4912/4913/4914 | IID/IIC/IIB/IIA | mannose/fructose/sorbose | 35.1 |
| 4977 | IIBC | N-acetylglucosamine/Glucose | 33.1 |
| 4982/4983 | IIA/IIBC | N-acetylglucosamine/Glucose | 31.1 |
| 5012 | IIBC | beta-glucoside/sucrose | 34.5 |
| 5017/5019 | IIA/IIBC | N-acetylglucosamine/Glucose | 33.0 |

*Unknown: can not be decided through either method.

Table S2 the PTS II gene sets in *Clostridium acetobutylicum* ATCC 824

| Cac Number | Domain Structure | Subfamily | GC% |
| --- | --- | --- | --- |
| CAP0066/0067/0068 | IIAB/IIC/IID | Mannose/fructose/Sorbose | 37.3 |
| 0154/0156 | IIBC/IIA | Mannitol/fructose | 33.7 |
| 0233/0234 | IIA/IIBC | Fructose/mannitol | 36.2 |
| 0383/0384/0386 | IIA/IIB/IIC | Lactose/cellobiose | 35.1 |
| 0423 | IIABC | beta-glucoside/sucrose | 33.5 |
| 0532 | IIBC | Glucose/ N-acetylglucosamine | 36.4 |
| 0570 | IIABC | Glucose/ N-acetylglucosamine | 36.1 |
| 1353/1354 | IIBC/IIA | N-acetylglucosamine/Glucose | 33.4 |
| 1407 | IIABC | beta-glucoside/sucrose | 34.3 |
| 1457/1458/1459/1460 | IIA/IIB/IIC/IID | Mannose/fructose/Sorbose | 33.9 |
| 2956/2957/2958 | IIC/IIB/IIA | Unknown | 31.2 |
| 2964/2965 | IIBC/IIA | Lactose/cellobiose | 33.4 |
| 2995 | IIA | Unknown | 31.2 |
| 3425/3427 | IIBC/IIA | Unknown | 32.5 |

Table S3 the PTS II gene sets in *Clostridium perfringens* str. 13

| Cpe Number | Domain Structure | Subfamily |
| --- | --- | --- |
| 0076 | IIC | Unknown |
| 0196 | IIBC | N-acetylglucosamine/Glucose |
| 0320/0321/0322/0323 | IIA/IIB/IIC/IID | Mannose/fructose/Sorbose |
| 0419 | IIBC | N-acetylglucosamine/Glucose |
| 0521/0522/0523 | IIA/IIB/IIC | Unknown |
| 0561 | IIBC | N-acetylglucosamine/Glucose |
| 0584 | IIABC | Fructose/mannitol |
| 0821/0822/0823/0824 | IIAB/IIC/IID/IID | Mannose/fructose/Sorbose |
| 1463/1464/1465/1466 | IID/IIC/IIB/IIA | Mannose/fructose/Sorbose |
| 1534 | IIBC | beta-glucoside/sucrose |
| 2157 | IIBC | N-acetylglucosamine/Glucose |
| 2229 | IIB | Unknown |
| 2577 | IIA | Lactose/cellobiose |
| 2629/2630/2631/2632 | IIA/IID/IIC/IIB | Mannose/fructose/Sorbose |

Table S4 the PTS II gene sets in *Clostridium tetani* E88

| Ctc Number | Domain Structure | Subfamily |
| --- | --- | --- |
| 0278 | IIABC | N-acetylglucosamine/Glucose |
| 1841 | Unknown | Unknown |

Table S5 the PTS II gene sets in *Clostridium difficile* 630

| Cd Number | Domain Structure | Subfamily |
| --- | --- | --- |
| 0041/0042/0043 | IIA/IIB/IIC | Unknown |
| 0135/0136/0137 | IIA/IIB/IIC | Lactose/cellobiose |
| 0206/0207/0208 | IIA/IIC/IIB | Fructose/mannitol |
| 0284/0285 | IIA/IIB | Mannose/fructose/Sorbose |
| 0286/0287/0288/0289 | IIA/IIB/IIC/IID | Mannose/fructose/Sorbose |
| 0388 | IIABC | beta-glucoside/sucrose |
| 0469 | IIABC | beta-glucoside/sucrose |
| 0491/0492/0493/0494 | IIA/IIB/IIC/IID | Mannose/fructose/Sorbose |
| 0764/0765/0766/0767 | IIC2/IIB/IIC/IIA | glucitol/sorbitol |
| 0813 | IIB | Unknown |
| 0816 | IIABC | beta-glucoside/sucrose |
| 0861/0862/0863 | IIB/IIC/IIA | Lactose/cellobiose |
| 1074/1076/1077/1078 | IIA/IIB/IIC/IID | Mannose/fructose/Sorbose |
| 1083 | IIB | Unknown |
| 1336 | IIBC | N-acetylglucosamine/Glucose |
| 1602 | IIB | Unknown |
| 2256 | IIC | Unknown |
| 2269 | IIBC | Fructose/mannitol |
| 2280/2281/2282 | IIC/IIB/IIA | Unknown |
| 2325/2326/2327 | IIC/IIB/IIA | Galactitol/? |
| 2332/2334 | IIA/IIBC | Fructose/mannitol |
| 2414/2417/2418 | IIA/IIBC/IIC2 | Glucitol/sorbitol |
| 2486/2487/2488 | IIC/IIB/IIA | Fructose/mannitol |
| 2510/2512 | IIBC/IIA | N-acetylglucosamine/Glucose |
| 2553/2555 | IIC/IIB | Unknown |
| 2566/2567/2568 | IIA/IIB/IIC | Fructose/mannitol |
| 2666/2667 | IIA/IIBC | N-acetylglucosamine/Glucose |
| 2880/2883/2884 | IIA/IIC/IIB | Lactose/cellobiose |
| 3013/3014/3015 | IIC/IIB/IIA | Fructose/mannitol |
| 3027/3030 | IIA/IIBC | N-acetylglucosamine/Glucose |
| 3048/3049 | IIC/IIB | Lactose/cellobiose |
| 3058/3061 | IIA/IIBC | N-acetylglucosamine/Glucose |
| 3067/3068/3069/3070 | IIA/IIB/IID/IIC | Mannose/fructose/Sorbose |
| 3075 | IIABC | Fructose/mannitol |
| 3080/3081/3082 | IIA/IIC/IIB | Lactose/cellobiose |
| 3086 | IIABC | Fructose/mannitol |
| 3089 | IIBC | Unknown |
| 3097 | IIABC | beta-glucoside/sucrose |
| 3101/3103 | IIBC/IIA | N-acetylglucosamine/Glucose |
| 3116 | IIABC | beta-glucoside/sucrose |
| 3125 | IIABC | beta-glucoside/sucrose |
| 3127 | IIBC | Unknown |
| 3132 | IIA | Fructose/mannitol |
| 3134 | IIABC | Fructose/mannitol |
| 3137 | IIABC | beta-glucoside/sucrose |
| 3276/3277/3278/3279 | IID/IIC/IIA/IIB | Mannose/fructose/Sorbose |
| 3443/3444/3445 | IIC/IIB/IIA | Lactose/cellobiose |
| 3629/3630 | IIC/IIB | Lactose/cellobiose |
| 3645/3647/3648 | IIC/IIA/IIB | Lactose/cellobiose |

Table S6 the PTS II gene sets in *Bacillus subtilis subsp. subtilis* str. 168

| Bsu Number | Domain Structure | Subfamily | GC% |
| --- | --- | --- | --- |
| 02350 | IICBA | N-acetylglucosamine/Glucose | 48.4 |
| 03980 | IICBA | Fructose/mannitol | 47.0 |
| 07700 | IICB | N-acetylglucosamine/Glucose | 47.4 |
| 07800 | IIBC | N-acetylglucosamine/Glucose | 48.1 |
| 08200 | IICB | N-acetylglucosamine/Glucose | 47.9 |
| 12010 | IICBA | Fructose/mannitol | 46.7 |
| 13890 | IICBA | N-acetylglucosamine/Glucose | 45.4 |
| 14400 | IIABC | Fructose/mannitol | 47.2 |
| 27040/27050/27060/27070 | IID/IIC/IIB/IIA | Mannose/fructose/Sorbose | 44.4 |
| 38050 | IIBC | beta-glucoside/sucrose | 47.2 |
| 38570/38580/38590 | IIA/IIC/IIB | Lactose/cellobiose | 49.9 |
| 39270 | IIBCA | beta-glucoside/sucrose | 50.8 |

Overall: 43.5%

Table S7 the PTS II gene sets in *Escherichia coli str. K-12* substr. DH10B

| ECDH10B Number | Domain Structure | Subfamily | GC% |
| --- | --- | --- | --- |
| 109 | IIA | Mannose/fructose/Sorbose | 49.2 |
| 744 | IICBA | N-acetylglucosamine/Glucose | 54.8 |
| 797 | IIABC | Fructose/mannitol | 54.6 |
| 1173 | IIBC | N-acetylglucosamine/Glucose | 52.7 |
| 1754 | IICB | N-acetylglucosamine/Glucose | 52.7 |
| 1874/1875/1876 | IIA/C/B | Lactose/cellobiose | 48.4 |
| 1955/1956/1957 | IIAB/C/D | Mannose/fructose/Sorbose | 51.6 |
| 2245/2246/2247 | IIC/B/A | Lactose/cellobiose | 48.3 |
| 2324 | IIBC | Fructose/mannitol | 55.7 |
| 2548/2551/2552 | IIA/C/B | Fructose/mannitol | 54.8 |
| 2582 | IIA | N-acetylglucosamine/Glucose | 47.3 |
| 2594 | IICB | N-acetylglucosamine/Glucose | 55.6 |
| 2870/2871/2872 | IIC/B/A | glucitol/sorbitol | 53.7 |
| 2883 | IIBC | N-acetylglucosamine/Glucose | 56.1 |
| 3306/ | IIB | Mannose/fructose/Sorbose | 51.2 |
| 3311/3312/3313 | IIB/C/D | Mannose/fructose/Sorbose | 49.0 |
| 3378 | IIA | Unknown | 52.2 |
| 3780 | IIABC | Fructose/mannitol | 53.9 |
| 3909 | IIABC | beta-glucoside/sucrose | 51.2 |
| 4089 | IIBC | Fructose/mannitol | 55.9 |
| 4136/4137/4138 | IIA/C/B | Fructose/mannitol | 54.6 |
| 4141 | IIB | Fructose/mannitol | 56.1 |
| 4388/4389/4390 | IIC/B/A | Lactose/cellobiose | 53.2 |
| 4435 | IIBC | N-acetylglucosamine/Glucose | 54.4 |
| 4506 | IIB | Lactose/cellobiose | 48.4 |
